# Assessment of Safety and Therapeutic Efficacy of *Rosa damascena* L. and *Quercus infectoria* on Cardiovascular Performance of Normal and Hyperlipidemic Rabbits: Physiologically Based Approach

**DOI:** 10.1155/2013/769143

**Published:** 2013-09-12

**Authors:** Siyavash Joukar, Masoumeh Askarzadeh, Beydolah Shahouzehi, Hamid Najafipour, Hossein Fathpour

**Affiliations:** ^1^Physiology Research Center, Kerman University of Medical Sciences, Kerman, P.O.Box 7616914115, Iran; ^2^Department of Physiology and Pharmacology, School of Medicine, Kerman University of Medical Sciences, Kerman, P.O. Box 7616914115, Iran; ^3^Department of Biology, Shahrekord Branch, Islamic Azad University, Shahrekord, Iran

## Abstract

According to the use of *Quercus infectoria* (QI) and *Rosa damascena* L. (RD) for therapeutic purposes and lack of adequate information about their cardiovascular effects, we investigated the cardiovascular indices of rabbits which chronically pretreated with these agents. Animal groups were control group (CTL), RD and QI groups with normal chow plus 1.5 g RD and QI extracts, respectively, in each kg of the diet for 45 days; Hyperlipidemic (H) group received high-fat diet for 45 days; H+RD and H+QI groups received high fat diet plus QI and RD extracts, respectively. Blood pressure was greater in H+RD group than CTL, RD, and H groups. Left ventricular developed pressure and left ventricular systolic pressure increased significantly in H+RD group versus CTL and RD groups (*P* < 0.05 and *P* < 0.0001, resp.) and in H+QI groups (*P* < 0.01 versus QI groups). Left ventricular end diastolic pressure (LVEDP) showed significant reduction in H+QI group versus H group. QI attenuated the values of total cholesterol, LDL, TG, and atherogenic indices of plasma when coadministrated with a high-fat diet. The results suggest the antilipidemic and antiatherogenic effects of QI. In addition, the use of RD along with a high-fat diet may increase the risk of hypertension in rabbits.

## 1. Introduction

Cardiovascular diseases (CVDs) account for about 30 percent of all deaths worldwide [1], and hyperlipidemia, hypertension, and smoking are well known as the three major risk factors of the mortality rates of CVDs [2]. Regarding the advances in the study and understanding of the mechanisms involved in the positive and negative effects of botanical drugs on health and diseases, one of the areas which has gained attention in recent years is the protective and destructive effects of herbal agents on the cardiovascular system.

Based on the previous experimental studies and existing traditional and folk medicine knowledge about some of the cardiovascular beneficial effects concerning *Rosa damascena* L. (RD) and *Quercus infectoria* (QI), we have selected these two herbal drugs for the present study. The *Q. infectoria* Olivier (Fagaceae) is a shrub that grows in Asia Minor, Iran, and Greece. The galls of *Q. infectoria* have analgesic [3] CNS depressant, antiparkinsonian, antidiabetic [3–5], anti-inflammatory [6], and antioxidant activity [7]. Recently, the hepatoprotective effects of *Q. infectoria* galls against CCL4-induced tissue damage have been reported [8]. *Rosa damascena* L. is a small plant, that is cultivated all over the world due to its scent and visual beauty [9]. The theraputical effects of *Rosa damascena* L. are due to its anti-inflammatory [10], analgesic, hypnotic, and antispasmodic [9, 11] properties. Antioxidant and antidiabetic [12, 13], heart inotropic [14], usefulness in treatment of menstrual bleeding [15], antitussive [16], tracheal relaxant [17], and relaxing activity [18] are the other effects that attributed to *Rosa damascena* L. 

Considering the beneficial effects of *Quercus infectoria* and *Rosa damascena* L. reported in the literature, the administration of these agents especially in the eastern societies is growing. In the present study we investigated the effects of chronic administration of *Quercus infectoria* and *Rosa damascena* L. on the hemodynamic, heart performance, lipid profile, and plasma atherogenic indices of rabbits with/without hyperlipidemia to elucidate the outcome of a long-term consumption of these agents on the cardiovascular system.

## 2. Material and Methods

Experiments were conformed to the national guidelines for conducting animal studies (Ethic committee permission no. 86/123KA—Kerman University of Medical Sciences, Iran) and were performed on 36 New Zealand White rabbits weighing between 2.5 and 3.5 kg. 

### 2.1. Materials

Sodium thiopental was purchased from Biochemie (Austria) and cholesterol from Merck (Germany). Galls of *Quercus infectoria* (QI) and flowers of *Rosa damascena* L. (RD) were collected during the spring of 2012 from Isfahan and Kerman (provinces of Iran), respectively; they were identified and confirmed by the Botany Department of Bahonar, University of Kerman, Iran. The galls of QI and air-dried flowers of RD (300 g) were grinded and macerated in 1000 mL methanol at room temperature for 3 days. Then the mixtures were filtered and evaporated in vacuum to yield a waxy mass extract from RD and a powder mass extract from QI [19].

### 2.2. Animals Groups and Experimental Protocol

Rabbits were kept under appropriate animal care and were randomly divided into 6 groups as control (CTL), RD, QI, Hyperlipidemic (H), Hyperlipidemic+RD (H+RD), and Hyperlipidemic+QI (H+QI). The CTL group was fed with normal rabbit chow. The RD and QI groups were fed with normal rabbit chow supplemented with 1.5 g RD and QI extracts, respectively, in each kg of the diet for 45 days. This dosage was calculated based on the current using pattern among consumers and previous studies [19]. Cholesterol (0.5%) and hydrogenated vegetable oil (16%) were added to the diet of the Hyperlipidemic (H) groups during the 45 days of this experiment. The H+QI and H+RD groups received 1.5 g QI and RD extracts, respectively, in each kg of their diet in addition to cholesterol and hydrogenated vegetable oil during the study [19]. The fasting blood sample was taken from ear vein on the first and the 46th day of the experiment in order to measure the plasma total cholesterol (TC), low-density lipoprotein (LDL), high-density lipoprotein (HDL), and the triglyceride (TG) levels by routine laboratory methods. The atherogenic indices of plasma, as markers of plasma atherogenicity, were calculated as TC/HDL [20] and LDL/HDL [21]. At the end of the experiment, animals were anaesthetized by the injection of sodium thiopental (50 mg/kg, ip) and were maintained with a 1% halothane in a 30% O_2_—69% N_2_O mixture during the surgical procedure. Deep anesthesia was confirmed and maintained throughout the surgery as judged by the absence of withdrawal response to a pinch stimulus applied to the hind limbs. The trachea was cannulated with spontaneous breathing throughout the experiment. A heparinized saline-filled (7 units/mL) cannula was connected to a pressure transducer, and a PowerLab analog to digital converter (AD Instruments, Australia) was inserted into the left carotid artery to record the heart rate and arterial blood pressure (BP). The other cannula which went through the right carotid artery was inserted into the left ventricle, and the left ventricular pressure (LVP) was recorded. The gaseous anesthesia was discontinued at the end of the surgery, and the time window for animal recovery from the surgery was 30 min. The mean arterial pressure (MAP) was calculated by “MAP = Pd + (Ps − Pd)/3 formula,” where Pd is the diastolic arterial pressure and Ps is the systolic arterial pressure. The maximum velocity of contraction (max dp/dt) and the maximum velocity of relaxation (min dp/dt) were calculated from the left ventricular pressure pulse [22]. Pressure-rate product (PRP), an indirect measure of myocardial oxygen demand, was determined as the product of the heart rate and mean arterial pressure ((MAP∗heart rate)∗ 1,000^−1^) [23].

### 2.3. Statistical Analysis

The results were presented as mean ± S.E.M. Comparisons were performed between basal and final values in each group by Student's paired *t*-test and among the different groups by one-way ANOVA which was followed by the post hoc Tukey's test. *P* value < 0.05 was considered as statistically significant.

## 3. Results

### 3.1. Lipids Profile and Atherogenic Indices

**Figure 1:**
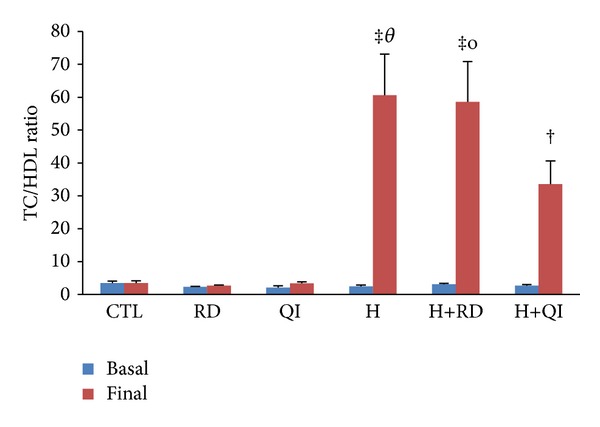
The basal and final values of plasma atherogenic index (total cholesterol to HDL cholesterol ratio, TC/HDL ratio) of the animal groups. The values are Mean ± SEM. *n* = 6; CTL: control; RD: *Rosa damascena* L.; QI: *Quercus infectoria*; H: Hyperlipidemic; H+RD: Hyperlipidemic+RD; H+QI: Hyperlipidemic+QI. ^‡^ *P* < 0.001 compared with the related basal value; ^*θ*^ *P* < 0.001 compared with the final values of the CTL, RD, and QI groups, ^o^ *P* < 0.001 compared with the final values of the RD and CTL; ^†^ *P* < 0.05 compared with the related basal value.

**Figure 2:**
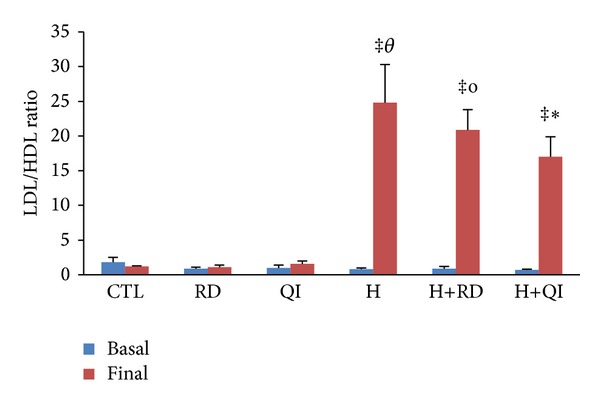
The basal and final values of plasma atherogenic index (LDL/HDL ratio) of the animal groups. The values are Mean ± SEM. *n* = 6, CTL: control; RD: *Rosa damascena* L.; QI: *Quercus infectoria*; H: Hyperlipidemic; H+RD: Hyperlipidemic+RD; H+QI: Hyperlipidemic+QI. ^‡^ *P* < 0.001 compared with the related basal value; ^o^ *P* < 0.001 compared with the final values of CTL, RD, and QI groups; ^*θ*^ *P* < 0.001 compared with the final values of the RD and CTL; ^‡^ *P* < 0.01 compared with the final values of the CTL and QI groups.

The levels of basal plasma lipids and basal atherogenic indices of the different groups had no significant difference. Consumption of normal chow alone or along with RD or QI for 45 days did not cause any significant change on the lipid profile and atherogenic indices of the CTL and QI groups. The triglyceride level, however, showed significant increase in the RD group (*P* < 0.01). The high-fat diet induced hyperlipidemia as significant increase in the TC, LDL, and TG and atherogenic indices in all groups compared to its related basal values (Table 1, Figures 1 and 2). The QI administration along with the hyperlipidic diet decreased the TC and LDL when compared to the H and H+RD groups (*P* < 0.001 and *P* < 0.01, resp.). The level of LDL was also reduced in the H+RD group (*P* < 0.01 versus H group). The level of TG was enhanced in the H group compared with the CTL group (*P* < 0.01); however, the consumption of QI attenuated this effect (*P* < 0.05 versus H group) (Table 1). Two atherogenic indices of plasma, TC/HDL and LDL/HDL, significantly increased in the H and H+RD groups (*P* < 0.001 versus CTL and RD groups). TC/HDL showed nonsignificant increase in the H+QI group (*P* < 0.055 compared to QI group and *P* = 0.058 compared to the CTL group) (Figure 1). The LDL/HDL ratio was associated with a lesser increase in the H+QI group than that of the H and H+RD (*P* < 0.01 when compared to the CTL and QI groups) (Figure 2).

### 3.2. Hemodynamic and Heart Functions

**Figure 3:**
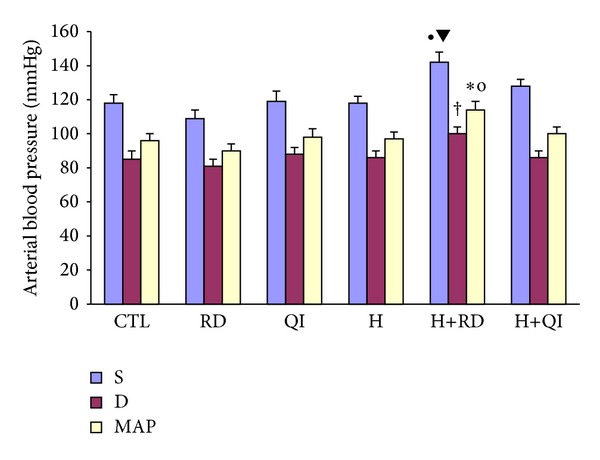
The arterial blood pressure in the different groups. The values are Mean ± SEM. *n* = 6; S: systolic pressure; D: diastolic pressure; MAP: mean arterial pressure; CTL: control; RD: *Rosa damascena* L.; QI: *Quercus infectoria*; H: Hyperlipidemic; H+RD: Hyperlipidemic+RD; H+QI: Hyperlipidemic+QI. ^•^ *P* < 0.05 versus the H and CTL groups; ^▾^ *P* < 0.001 versus the RD group, ^†^ *P* < 0.01 versus the RD group; *P < 0.05 versus the CTL; ^o^ *P* < 0.01 versus the RD group.

At the end of this study, the comparison of the blood pressure among the different animal groups did not show any significant effect of RD or QI (each alone) on this parameter. Yet, in those animals which received a combination of RD and high-fat diet, systolic, diastolic, and the mean arterial pressures increased significantly whenever it was compared to its corresponding groups, that is, the CTL, RD, and H. In this study, the high-fat regimen alone or plus QI had no significant effect on the blood pressure compared to its matching groups (Figure 3).

The heart rate, pulse pressure, the maximum velocity of heart contraction (+dp/dt max = max dp/dt), and the maximum velocity of heart relaxation (−dp/dt max= min dp/dt) did not show any significant difference among the animal groups (Table 2). The PRP was greater in the H+RD and H+QI groups; however, this index was only significant in the H+RD, when compared to the RD group (*P* < 0.05) (Table 2).

**Figure 4:**
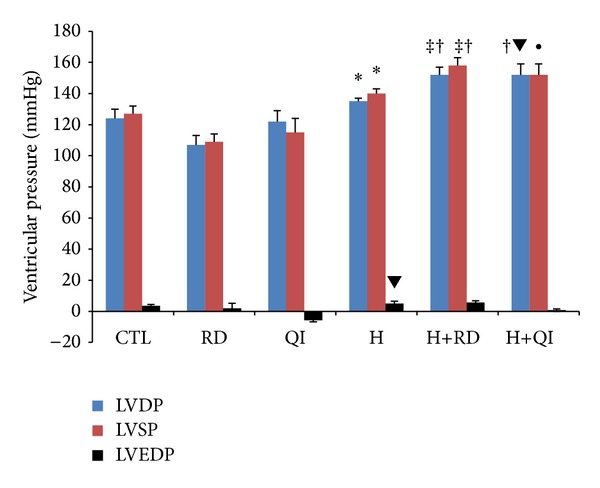
The left ventricular pressure in different groups. The values are Mean ± SEM. *n* = 6; LVDP: left ventricular developed pressure; LVSP: the left ventricular systolic pressure; LVEDP: left ventricular end diastolic pressure; CTL: control; RD: *Rosa damascena* L.; QI: *Quercus infectoria*; H: Hyperlipidemic; H+RD: Hyperlipidemic+RD; H+QI: Hyperlipidemic+QI. *P < 0.05 compared to CTL; ^‡^ *P* < 0.0001 compared with RD; ^†^ *P* < 0.05 compared with RD; ^▾^ *P* < 0.05 compared with QI; ^•^ *P* < 0.01 compared with QI.

The left ventricular developed pressure (LVDP) and the left ventricular systolic pressure (LVSP) increased significantly in the H+RD group compared to the CTL and RD groups (*P* < 0.05 and *P* < 0.0001, resp.) and in the H+QI groups (*P* < 0.01 versus QI groups). The LVDP and LVSP also increased in the H groups compared to the RD group (*P* < 0.05). The H and the H+RD groups showed maximum levels of left ventricular end diastolic pressure (LVEDP); on the contrary, the QI group showed a minimum level of this parameter. In addition, the LVEDP had dropped in the H+QI group compared to the H group (*P* < 0.05) (Figure 4). 

## 4. Discussion

This study aimed to assess the influence of the chronic administration of a methanolic extract of two famous herbal drugs, that is, *Rosa damascena* L. and *Quercus infectoria*, on hemodynamic, heart performance, lipid profile, and plasma atherogenic indices of rabbits with/without high-fat diet.

The results revealed the obvious beneficial effect of QI on harmful outcomes of a hyperlipidic diet as the attenuation of plasma atherogenic indices, the prevention of hyperlipidemia, and the improvement of the cardiovascular performance. On the other hand, the RD administration showed a mild decreasing effect on plasma lipid profile and the atherogenic indices. However, the administration of RD along with a high-fat diet increased the index of myocardial oxygen consumption and the risk of hypertension. 

The inhibition of pancreatic lipase (PL) as a pivotal enzyme in the intestinal absorption of triglycerides and HMG CoA reductase, the other important enzyme, that is, involved in the endogenous cholesterol biosynthesis, is the target of the PL inhibitors [24] and statins, respectively [25]. In vitro experiments indicated that especially QI and to some extent RD have inhibitory effect on pancreatic lipase [26] and HMG CoA reductase enzyme [27]. The results of previous [19] and present in vivo studies obviously have confirmed the antilipidemic effect of QI, but RD had no considerable effect. Therefore, a part of the antilipidemic and antiatherogenic effects of QI revealed in the present study may mediate through inhibition of PL and HMG CoA reductase enzymes. In addition, QI contains some bioactive agents, and its main component is tannin [8]. This phenolic compound is able to precipitate proteins [28, 29], for example, PL enzyme, and hence may provide a portion of antilipidemic and antiatherogenic effects of QI. 

Previous studies showed that the use of high-lipid diet contains 1% cholesterol for 8 [30] and 10 weeks [31] and had no significant effect on the blood pressure, heart rate, PRP, max dp/dt, and min dp/dt indices of rabbits. Still, even 4 weeks of a high-cholesterol regimen increases the vascular resistance and decreases the endothelium-dependent vasodilatation [32]. Consistent with the previous reports, in our study, 45 days of a highfat diet had no significant effect on hemodynamic and heart performance of the H group compared to the CTL group. However, coadministration of RD and high-fat diet was associated with an increase in the blood pressure, LVSP, and LVDP. 

The positive chronotropic and inotropic effect of RD has been observed in the isolated heart of Guinea Pigs. This effect apparently is mediated through the stimulation of *β*-adrenoceptor [33, 34]. The opening of the calcium channels and the elevation of the intracellular cAMP levels like the effect of phosphodiesterase III inhibitors [35] are likely to be the other possible mechanisms which may be involved in the inotropic effect of RD.

Blood pressure is determined by product of cardiac output and peripheral vascular resistance. Cardiac output, in turn, is the product of stroke volume and heart rate. The increase of heart contractility leads to the increase of stroke volume and consequently the blood pressure [36]. In our study, a nonsignificant increasing trend of max dp/dt as an index of heart contractility in the H+RD group was observed. However, we did not observe increase in heart rate in presence of RD. The discrepancy between our findings and the results of Boskabady et al. [33] related to the chronotropic effect of RD may come from the two different conducting methods, in vivo method versus isolated heart method, and hence influence of endogenic factors such as autonomic nervous system feedback in our study. The combination of the negative effect of hyperlipidemia on the vascular vasodilatation along with the partial increase of heart contractility due to positive inotropic effect of RD is a likely reason for explaining the high blood pressure and LVSP in the H+RD group. However, there is the possibility of unknown effects of RD on the arterial vessel that should be investigated in future studies. 

## 5. Conclusions

 The results of this study revealed the significant antilipidemic and antiatherogenic effects of QI but not RD. This may be partly mediated by the inhibition of PL and HMG CoA reductase enzymes. Regarding some side effects of synthetic lipid-lowering drugs, for example, myopathy and liver damages for statins [25, 37] and gastric irritation, flushing, hyperuricemia, dry skin, and abnormal liver function for PL synthetic inhibitors [38], QI can be considered as a new candidate for reducing plasma lipids in future human studies. In addition, the use of RD along with a high-fat diet increased the risk of hypertension in rabbits. If our results can be extrapolated to human, this adverse effect of RD in cases with hyperlipidemia context should be considered and investigated. 

## Figures and Tables

**Table 1:** Plasma lipids levels in different animal groups.

Groups	Basal values				Final values			
	TC (mg/dL)	HDL (mg/dL)	LDL (mg/dL)	TG (mg/dL)	TC (mg/dL)	HDL (mg/dL)	LDL (mg/dL)	TG (mg/dL)
CTL	50.5 ± 2.8	16 ± 2.2	23.2 ± 4.2	86.2 ± 4.6	56 ± 5.2	18 ± 2.5	20.5 ± 3.2	90.7 ± 5.2
RD	38 ± 5.5	18.1 ± 3.2	16 ± 3.8	75.7 ± 7.8	49.6 ± 11.4	20.4 ± 5.8	16.5 ± 5	152.6 ± 14.6^‡^
QI	55.8 ± 22.7	23.4 ± 5.2	30.2 ± 17.3	75.5 ± 4.9	70.4 ± 18	20.2 ± 5	32 ± 15.1	153.8 ± 41
H	48 ± 3.8	20.4 ± 2.9	16.2 ± 5.9	77.2 ± 7.4	1328 ± 62.2^‡‡a^	24.8 ± 3.5	536 ± 19^‡‡e^	211 ± 23^‡g^
H+RD	59.8 ± 6.7	19.7 ± 1.7	15.7 ± 4.4	82.2 ± 9.4	1088 ± 168^‡a^	20 ± 1.7	395 ± 29.7^‡‡f^	142.7 ± 17.7^†^
H+QI	52 ± 5	20.2 ± 2.1	15 ± 4.2	73 ± 11.2	642 ± 91.8^‡a,b,c^	21.3 ± 2.8	325 ± 29.1^‡f^	115.8 ± 20.8^†h^

Values are Mean ± SEM. *n* = 6; ^‡‡^ *P* < 0.001 compared with related basal value; ^‡^ *P* < 0.01 compared with related basal value; ^†^ *P* < 0.05 compared with related basal value; ^a^ *P* < 0.001 compared with final values of CTL, RD, and QI; ^b^ *P* < 0.001 compared with final value of H; ^c^ *P* < 0.01 compared with final value of H+RD; ^e^ *P* < 0.001 compared with final values of CTL, RD, QI, H+RD, and H+QI; ^f^ *P* < 0.001 compared with final values of CTL, RD, QI, and H; ^g^ *P* < 0.01 compared with final value of CTL; ^h^ *P* < 0.05 compared with final value of H. CTL: control; RD: *Rosa damascena* L.; QI: *Quercus infectoria*; H: Hyperlipidemic; H+RD: Hyperlipidemic+RD; H+QI: Hyperlipidemic+QI; TC: plasma total cholesterol; LDL: low-density lipoprotein; HDL: high-density lipoprotein; TG: triglyceride.

**Table 2:** Pulse pressure and cardiac indices in different animal groups.

Groups	Pulse pressure (mmHg)	Max dp/dt (mmHg/s)	Min dp/dt (mmHg/s)	HR (beat/min)	PRP
CTL	33 ± 5	4586 ± 292	−2698 ± 71	291 ± 13	28 ± 2
RD	28 ± 4	4554 ± 306	−2746 ± 234	247 ± 7	22.4 ± 1.2
QI	32 ± 3	4540 ± 667	−3648 ± 665	277 ± 16	27.3 ± 2.4
H	32 ± 2	4877 ± 324	−2734 ± 217	274 ± 11	26.5 ± 1.4
H+RD	42 ± 2	5689 ± 946	−3618 ± 254	276 ± 8	31.6 ± 2.1*
H+QI	42 ± 2	5534 ± 532	−3765 ± 406	302 ± 19	30.4 ± 2.9

Values are Mean ± SEM. *P < 0.05 compared with RD. CTL: control; RD: *Rosa damascena* L.; QI: *Quercus infectoria*; H: Hyperlipidemic; H+RD: Hyperlipidemic+RD; H+QI: Hyperlipidemic+QI; PRP: Pressure-rate product ((MAP ∗ heart rate) ∗ 1,000^−1^); max dp/dt: maximum velocity of contraction; min dp/dt: maximum velocity of relaxation.
